# Supplementary material for: Tead1a Initiates Transcriptional Priming Through the TEAD1a/YAP‐Notch1‐Spi1/Cebpα Axis to Promote Neutrophil Fate
Source: Adv Sci (Weinh). 2025 Aug 28;12(41):e05441. doi: 10.1002/advs.202505441 (PMC12591127; doi:10.1002/advs.202505441)
Supplement: Supplementary file 4 — Supplementary Dataset [file ADVS-12-e05441-s004.zip › 3-8.zip!/3-8/0904/0904/05.annot/Neutrophils/dotplot.pdf]

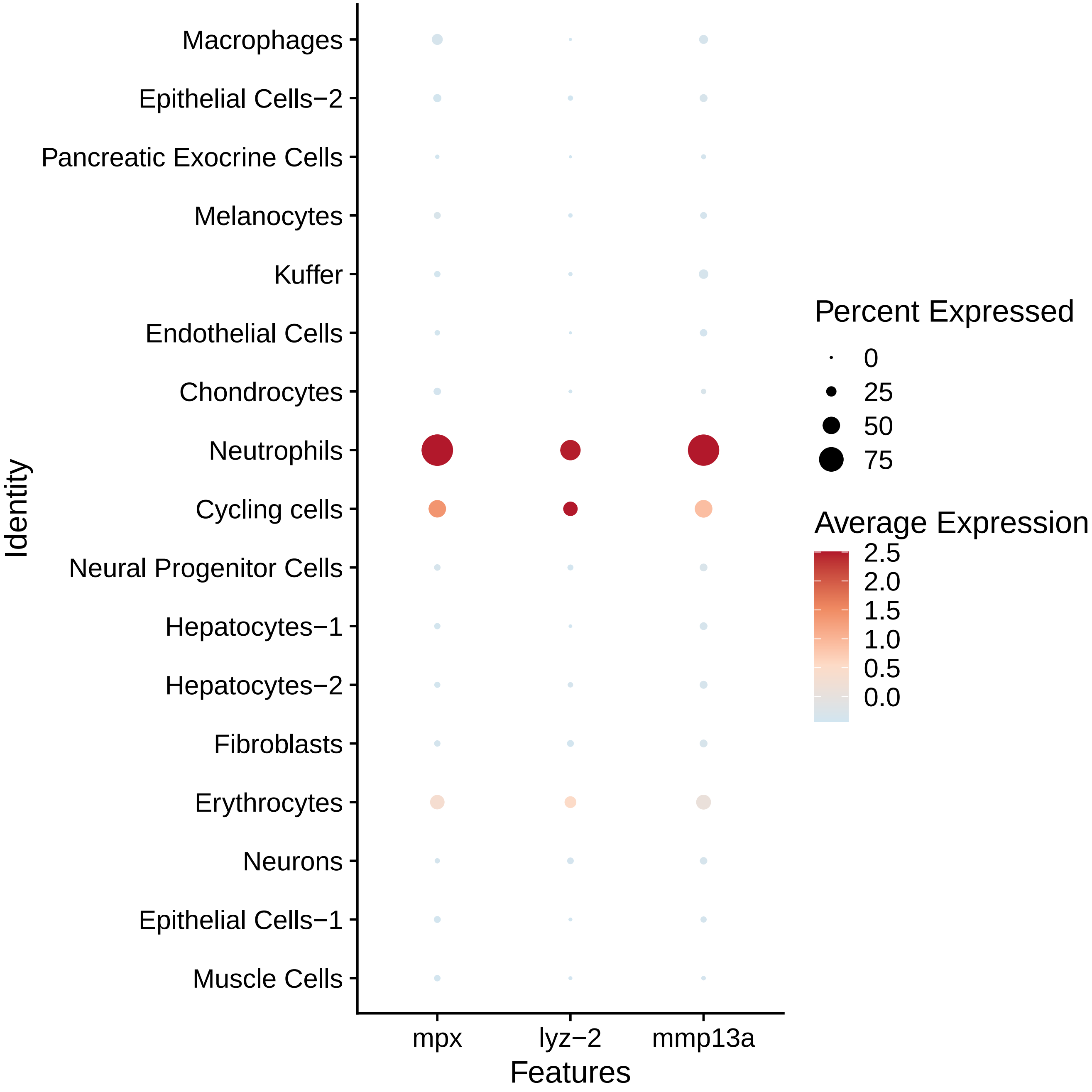

Macrophages
Epithelial Cells−2
Pancreatic Exocrine Cells
Melanocytes
Kuffer
Endothelial Cells
Chondrocytes
Neutrophils
Cycling cells
Neural Progenitor Cells
Hepatocytes−1
Hepatocytes−2
Fibroblasts
Erythrocytes
Neurons
Epithelial Cells−1
Muscle Cells
Identity
mpx
lyz−2
mmp13a
Features
Percent Expressed
0
25
50
75
Average Expression
2.5
2.0
1.5
1.0
0.5
0.0
